# Exercise intensity prescription in cancer survivors: ventilatory and lactate thresholds are useful submaximal alternatives to VO_2peak_

**DOI:** 10.1007/s00520-020-05407-y

**Published:** 2020-03-16

**Authors:** Justine Schneider, Kathrin Schlüter, Tanja Sprave, Joachim Wiskemann, Friederike Rosenberger

**Affiliations:** 1 Working Group Exercise Oncology, Department of Medical Oncology, National Center for Tumor Diseases (NCT), Heidelberg University Hospital, Im Neuenheimer Feld 460, 69120 Heidelberg, Germany; 2 Institute of Sports and Sport Science, Heidelberg University, Heidelberg, Germany; 3 Department of Radiation Oncology, University Hospital Heidelberg and National Center for Radiation Oncology (NCRO), Heidelberg Institute for Radiation Oncology (HIRO), Heidelberg, Germany; 4 Division of Health Sciences, German University of Applied Sciences for Prevention and Health Management (DHfPG), Saarbrücken, Germany

**Keywords:** Aerobic training, Endurance training, Oncology, Peak oxygen uptake, Lactate threshold, Ventilatory threshold

## Abstract

**Purpose:**

Most studies with cancer survivors use percentages of peak oxygen uptake (VO_2peak_) for intensity prescription. Lactate or ventilatory thresholds might be useful submaximal alternatives, but this has never been investigated. Therefore, we aimed at comparing three training sessions prescribed using %VO_2peak_ (reference), lactate thresholds, and ventilatory thresholds in terms of meeting the vigorous-intensity zone, physiological, and psychological responses.

**Methods:**

Twenty breast (58 ± 10 years) and 20 prostate cancer survivors (68 ± 6 years), 3.6 ± 2.4 months after primary therapy, completed a maximal cardiopulmonary exercise test and three vigorous training sessions in randomized order: 38 min of cycling at 70% VO_2peak_ (M-VO_2peak_), 97% of individual anaerobic lactate threshold (M-IAT), and 67% between ventilatory thresholds 1 and 2 (M-VT). Heart rate (HR), blood lactate concentration (bLa), perceived exertion, and enjoyment were assessed.

**Results:**

Cancer survivors exercised at 75 ± 23, 85 ± 18, and 79 ± 19 W during M-VO_2peak_, M-IAT, and M-VT (*p* > .05). Sessions could not be completed in 3, 8, and 6 cases. Session completers showed HR of 82 ± 7, 83 ± 9, and 84 ± 8 %HR_peak_ and bLa of 3.7 ± 1.9, 3.9 ± 0.9, and 3.9 ± 1.5 mmol·l^−1^, which was not different between sessions (*p* > .05). However, variance in bLa was lower in M-IAT compared to M-VO_2peak_ (*p* = .001) and to M-VT (*p* = .022).

**Conclusion:**

All intensity prescription methods on average met the targeted intensity zone. Metabolic response was most homogeneous when using lactate thresholds.

**Implications for cancer survivors:**

Submaximal thresholds are at least as useful as VO_2peak_ for intensity prescription in cancer survivors. Overall, slightly lower percentages should be chosen to improve durability of the training sessions.

## Introduction

Exercise is recommended for cancer survivors by expert panels worldwide because of its various beneficial effects [1–4]. Today, it represents a recognized part of supportive therapy. Regarding aerobic activity, 150 min of moderate or 75 min of vigorous-intensity exercise or an equivalent combination per week is recommended [1, 2]. Meeting these zones of moderate or vigorous intensity is crucial to elicit the intended training effects and avoid underload or overload of patients.

Some expert panel recommendations for cancer survivors do not specify how to target different intensity zones [1]. Others suggest 60–85% of oxygen uptake reserve (VO_2_R) or 60–85% of heart rate reserve (HRR) or > 70% of peak heart rate (HR_peak_) to target the vigorous-intensity zone [5, 6]. However, percentages of VO_2_R are rarely used due to the effort of resting oxygen uptake measurements. Furthermore, an analysis of cardiopulmonary exercise tests (CPETs) showed that percentages of HRR result in an intensity overload in cancer survivors due to their elevated resting heart rate [6, 7], whereas percentages of HR_peak_ appear appropriate [6, 8]. Most studies in cancer survivors prescribe certain percentages of peak oxygen uptake (VO_2peak_) to target intensity zones [9, 10]. However, all relative percent concepts can be criticized for assuming that a fixed percentage of a reference value represents the same intensity in all individuals. These percentages might fit on average, but be imprecise on an individual level.

Individual threshold concepts, i.e., blood lactate (bLa) or ventilatory thresholds [11, 12], which are anchored to the individual metabolic profile, are considered more accurate than fixed percentages of reference values [13, 14]. Furthermore, their determination does not require maximal exhaustion which appears valuable in cancer survivors who are not able or willing to spend maximal effort. Threshold concepts have a long tradition in high-performance and recreational sports due to their capability of maximizing physical performance [14–16] and are also considered superior in maximizing beneficial effects of exercise in cancer survivors [6]. However, research on threshold concepts for intensity prescription in cancer survivors is scarce. So far, ventilatory thresholds have only been used for prescribing low to moderate but no vigorous-intensity exercise [17–19], whereas bLa thresholds have not been used for intensity prescription in cancer survivors yet. Their determination usually requires completing at least five stages of a stepwise incremental exercise protocol [20] which is not possible for many cancer survivors due to their low aerobic fitness [6, 21]. However, it appears worth testing whether bLa thresholds can be determined in a CPET protocol with 1-min stages [6, 21] and used for intensity prescription in cancer survivors.

Therefore, the present study aimed at comparing three vigorous training sessions in terms of durability, physiological, and psychological responses: (a) a session prescribed in percentages of VO_2peak_ which, as the most commonly used method, served as reference here; (b) a session prescribed by means of ventilatory thresholds; and (c) for the first time with cancer survivors, a session prescribed by means of bLa thresholds, all determined from one CPET. It was hypothesized that the threshold-based methods meet the vigorous-intensity zone as successfully as percentages of VO_2peak_ but elicit a more homogeneous metabolic response as they are anchored to the individual metabolic profile. Knowledge on this will improve exercise intensity prescription for cancer survivors.

## Methods

### Participants

A total of 40 cancer survivors, 20 with breast and 20 with prostate cancer to represent the most common types of cancer in females and males, were recruited for participation. Participants were recruited by means of advertising flyers at the breast and prostate cancer presentations of our comprehensive cancer center and medical practices, hospitals, and self-help groups around. All participants met the following inclusion criteria: diagnosed with breast or prostate cancer, 6 to 52 weeks after end of primary therapy (i.e., surgery and/or radiotherapy and/or chemotherapy), 18 to 75 years of age, and no regular vigorous endurance or resistance training (> 1 session per week) within the last 6 months. Exclusion criteria were diagnosis with additional other cancer or severe comorbidities that preclude participation in exercise testing or training (acute infectious diseases, severe cardiac, respiratory, renal, or neurological diseases).

### General design

Following a cross-sectional design, each patient performed four tests: a CPET and then three vigorous-intensity training sessions targeted by means of the three different prescription methods in randomized order (block randomization procedure). All tests took place once per week, separated by at least 4 days to avoid training adaptations, and were conducted on electromagnetically braked cycle ergometers (Ergoselect 100 or 200, Ergoline, Bitz, Germany).

### Cardiopulmonary exercise tests

CPETs were preceded by a 2-min resting period on the cycle ergometer. They started at 20 W and increased every minute by 10 W until volitional exhaustion. Patients were encouraged to exert maximal effort. After a 10-min rest following the CPET, each patient performed a supramaximal verification test. The protocol started at 20 W and work rate was rapidly manually increased to 110% peak power output (PPO) of the preceding CPET [22]. Patients were again encouraged to exert maximal effort and the test was continued until volitional exhaustion.

A 12-lead electrocardiogram was continuously monitored (CardioPart 12 Blue, Amedtec, Aue, Germany). Gas exchange data were continuously measured using a breath-by-breath gas analysis system (Ergostik, Geratherm Respiratory, Bad Kissingen, Germany). The system was calibrated prior to each test according to the manufacturer’s guidelines. For bLa determination, capillary blood samples from the hyperemized (Finalgon®) earlobe were taken at rest, at the end of each 1-min increment, and after exercise cessation. They were analyzed using an enzymatic-amperometric method (Super GL compact, Hitado, Möhnesee, Germany). Blood pressure (Bp) and ratings of perceived exertion (RPE, 6 to 20 BORG scale [23]) were assessed every 2 min.

PPO was interpolated when appropriate. VO_2peak_ and HR_peak_ were defined as the highest 20-s average value reached during or immediately after the CPET. Ventilatory threshold 1 (VT1) and 2 (VT2) were determined using the V-slope method (VCO_2_/VO_2_) [11] as primary and the VE/VCO_2_ method [16] as secondary criterion. The individual anaerobic bLa threshold (IAT) was determined at 1 mmol L^−1^ above minimum lactate equivalent (Ergonizer, Freiburg, Germany) [24]. This concept was originally designed for a 3-min exercise stage protocol. However, it was adapted here to the CPET protocol because cancer survivors’ fitness levels are usually not sufficient to receive an evaluable blood lactate curve when using 3-min exercise stage protocols.

CPETs were considered maximal when VO_2peak_ in the verification test did not exceed VO_2peak_ in the CPET by more than 3% (verification criterion). This verification criterion represents the measurement accuracy of VO_2_ determination reported by the manufacturer (Ergostik, Geratherm Respiratory, Bad Kissingen, Germany). Furthermore, following secondary criteria, CPETs were considered maximal when two or more of the following criteria occurred: maximal respiratory exchange ratio (RER_peak_) ≥ 1.1, HRpeak ≥ 200 minus age, peak bLa (bLa_peak_) ≥ 8 mmol L^−1^, RPE ≥ 18 [5, 25, 26]. However, VO_2peak_ from the CPET (irrespective of maximal or not) was used to derive training intensity because this is the usual procedure in literature that should serve as a reference here.

### Training sessions

Training sessions lasted 38 min to theoretically reach 75 min of vigorous-intensity exercise as recommended when performing two sessions per week [1]. All sessions were designed to target the vigorous-intensity zone: 70% VO_2peak_ [8, 27] (method VO_2peak_, M-VO_2peak_), slightly below (97%) IAT [15, 16, 20] (M-IAT), and two-thirds (67%) between VT1 and VT2 [11, 16] (M-VT). Power output (W) corresponding to these points was prescribed. To assess the evoked strain, HR (Polar A300 monitor, Polar Electro Oy, Kempele, Finland), bLa, Bp, and RPE were recorded at rest and after 10, 20, 30, and 38 min of exercise. Exercise values were averaged over the four measurement time points. A lactate steady state (LASS) was defined as an increase in bLa of ≤ 0.9 mmol L^−1^ during the last 18 min of each training session (≤ 0.05 mmol L^−1^ min^−1^) [15, 28]. Enjoyment was assessed after each training session using a single-item 7-point Likert scale (“How much did you enjoy the training session?” 1 = not at all to 7 = very much) adjusted from Rogers et al. [29]. For safety assessment, adverse events were recorded.

### Statistical analyses

The sample size was based on a preceding similar study with healthy male participants [30]. Normality was tested using the Shapiro-Wilk test. Differences between the three training sessions for continuous data were assessed by one-way repeated measures analysis of variance (ANOVA) or in the case of non-parametric or ordinal scaled data by Friedman’s ANOVA. Differences between cancer entity were calculated using independent *t* tests or the Mann-Whitney *U* test in the case of non-parametric or ordinal scaled data. Dependent dichotomous data were assessed using Cochran’s *Q* test with McNemar post hoc test. For independent dichotomous data, the *χ*^2^ test was used. The Pitman-Morgan test was used to test for differences of homogeneity of bLa and %HR_peak_ response between training sessions. Correction for multiple testing was applied using the Bonferroni-Holm post hoc test. *p* < .05 was considered significant. Data are presented as means ± standard deviations or individual courses. All Data were analyzed using IBM SPSS Version 25 (IBM Corp, Armonk, NY) and MATLAB Version R2018a (MathWorks, Natick, MA).

## Results

Participants’ characteristics are shown in Table 1. VO_2peak_ could not be determined in one case due to fear of wearing a facemask, IAT could not be determined in one case because of a near linear bLa curve, and thus, M-VO_2peak_ and M-IAT could not be performed in one case each. Seven out of the 39 CPETs with VO_2peak_ measurement (18%) were not considered maximal based on secondary criteria for maximal exhaustion. Interestingly, according to the verification test, these seven CPETs were all considered maximal, whereas 13 other CPETs did not satisfy the verification criterion (i.e., VO_2peak_ reached during the verification test was more than 3% higher than VO_2peak_ reached during CPET). In other words, according to the verification test, 33% of the performed CPETs were not considered maximal. Still, all patients were included in the data analyses, in order to reflect what is usually done in practice. One patient’s HR had to be excluded from data analyses as a result of measurement problems.

**Table 1:** Participants’ characteristics. Data presented as mean ± SD unless stated otherwise

	Total	BCa	PCa
*n*	40	20	20
Age	62.9 ± 9.2	58.4 ± 9.7	67.5 ± 6.0
BMI (kg/m^2^)	27.4 ± 3.9	27.1 ± 4.8	27.7 ± 2.7
Time since diagnosis (months)	20.8 ± 29.1	9.7 ± 3.5	32.0 ± 38.2
Time since end of primary treatment^†^ (months)	3.6 ± 2.4	3.5 ± 2.0	3.8 ± 2.7
Type of treatment received, *n* (%)			
Surgery	36 (90)	20 (100)	16 (80)
Chemotherapy	10 (25)	10 (50)	0 (0)
Radiation	32 (80)	18 (90)	14 (70)
Antihormonal therapy^‡^	23 (58)	17 (85)	6 (30)
Current ß-blocker intake, *n* (%)	11 (28)	5 (13)	6 (15)
VO_2peak_ (mL/min/kg), *n*	19.7 ± 4.1, 39	19.2 ± 3.4, 20	20.3 ± 4.7, 19
PPO (W/kg)	1.6 ± 0.4	1.5 ± 0.3	1.7 ± 0.4

*BMI*, body mass index; *BCa*, breast cancer patients; *PPO*, peak power output; *PCa*, prostate cancer patients; *SD*, standard deviation; *VO*_*2peak*_, peak oxygen consumption

^†^Surgery and/or radiotherapy and/or chemotherapy

^‡^21 of 23 participants were still undergoing antihormonal therapy at the beginning of the study

The results of the training sessions are presented in Table 2. Prescribed absolute power output did not differ between the training sessions (all *p* > .05), but prescribed relative power output was lower for M-VO_2peak_ when compared to M-IAT (*p* = .028) and to M-VT (*p* = .036). M-VO_2peak_, M-IAT, and M-VT were terminated prematurely in 3, 8, and 6 cases, respectively, which was not different between training sessions (*p* = .093). Sixteen of the 17 premature terminations were due to muscular exhaustion, whereas one resulted from knee pain, which was considered as a minor adverse event. No severe adverse event occurred. When comparing %PPO between session completers and those who terminated prematurely, there was no significant difference for M-VO_2peak_ (57 vs. 61 %PPO, *p* = .484), whereas %PPO of completers was significantly lower for M-IAT (62 vs. 72 %PPO, *p* = .006) and M-VT (58 vs. 70 %PPO, *p* = .001).

**Table 2:** Comparison of the three exercise sessions. Intensity during sessions was prescribed as follows: 70% VO_2peak_ (M-VO_2peak_), 97% IAT (M-IAT), and 67% between VT1 and VT2 (M-VT). Data presented as mean ± SD unless stated otherwise

		M-VO_2peak_	M-IAT	M-VT
Prescribed power output (W)	Total (*n* = 38)	75 ± 23	85 ± 18	79 ± 19
	BCa (*n* = 19)	64 ± 17*^†^	71 ± 9*^†^	70 ± 16*^†^
	PCa (*n* = 19)	86 ± 24*^†^	98 ± 16*^†^	88 ± 19*^†^
Prescribed relative power output (%PPO)	total (*n* = 38)	57 ± 9	64 ± 9*	61 ± 8*
	BCa (*n* = 19)	57 ± 10	62 ± 7	62 ± 9
	PCa (*n* = 19)	57 ± 9	66 ± 11	59 ± 8
Number of premature session terminations	Total (*n* = 38)	3	8	6
	BCa (*n* = 19)	2	3	4
	PCa (*n* = 19)	1	5	2
Relative heart rate (%HR_peak_)	Total (*n* = 28)^‡^	82 ± 7	83 ± 9	84 ± 8
	BCa (*n* = 14)	84 ± 6	84 ± 6	87 ± 6
	PCa (*n* = 14)	80 ± 7	83 ± 12	81 ± 8
Blood lactate concentration (mmol L^−1^)	Total (*n* = 28)^‡^	3.7 ± 1.9	3.9 ± 0.9	3.9 ± 1.5
	BCa (*n* = 14)	4.1 ± 1.8	4.2 ± 1.1	4.5 ± 1.1*^†^
	PCa (*n* = 14)	3.4 ± 2.0	3.6 ± 0.7	3.3 ± 1.6*^†^
Proportion of participants attaining LASS^§^ (%)	Total (*n* = 36, 31, 34)	92	97	91
	BCa (*n* = 18, 16, 16)	90	80	94
	PCa (*n* = 18, 15, 18)	83	93	89
RPE breathing (scale 6–20)	Total (*n* = 28)^‡^	12.7 ± 2.3	12.9 ± 1.8	12.8 ± 2.2
	BCa (*n* = 14)	13.4 ± 2.0	13.4 ± 2.0	13.6 ± 2.6*^†^
	PCa (*n* = 14)	11.9 ± 2.4	12.5 ± 1.5	12 ± 1.4*^†^
RPE legs (scale 6–20)	Total (*n* = 28)^‡^	12.8 ± 2.2	13.3 ± 2.1	13.0 ± 2.4
	BCa (*n* = 14)	13.4 ± 2.0	13.9 ± 2.4	13.9 ± 2.6*^†^
	PCa (*n* = 14)	12.3 ± 2.4	12.7 ± 1.5	12.1 ± 1.9*^†^
Enjoyment (scale 1–7)	Total (*n* = 28)^‡^	5.5 ± 1.4	5.5 ± 1.4	5.3 ± 1.3
	BCa (*n* = 14)	5.1 ± 1.7	5.3 ± 1.8	5.1 ± 1.5
	PCa (*n* = 14)	5.9 ± 0.9	5.8 ± 0.9	5.6 ± 0.9

*BCa*, breast cancer patients; *PCa*, prostate cancer patients; *LASS*, lactate steady state; *RPE*, rating of perceived exertion; *SD*, standard deviation

*Significant difference compared with M-VO_2peak_: p < 0.05

*^†^Significant difference between cancer entities: *p* < 0.05

^‡^Reduction from *n* = 38 to *n* = 28 due to exclusion of data from participants with early session terminations

^§^Proportion of participants who attained LASS among those who completed the respective exercise session

**Fig. 1:**
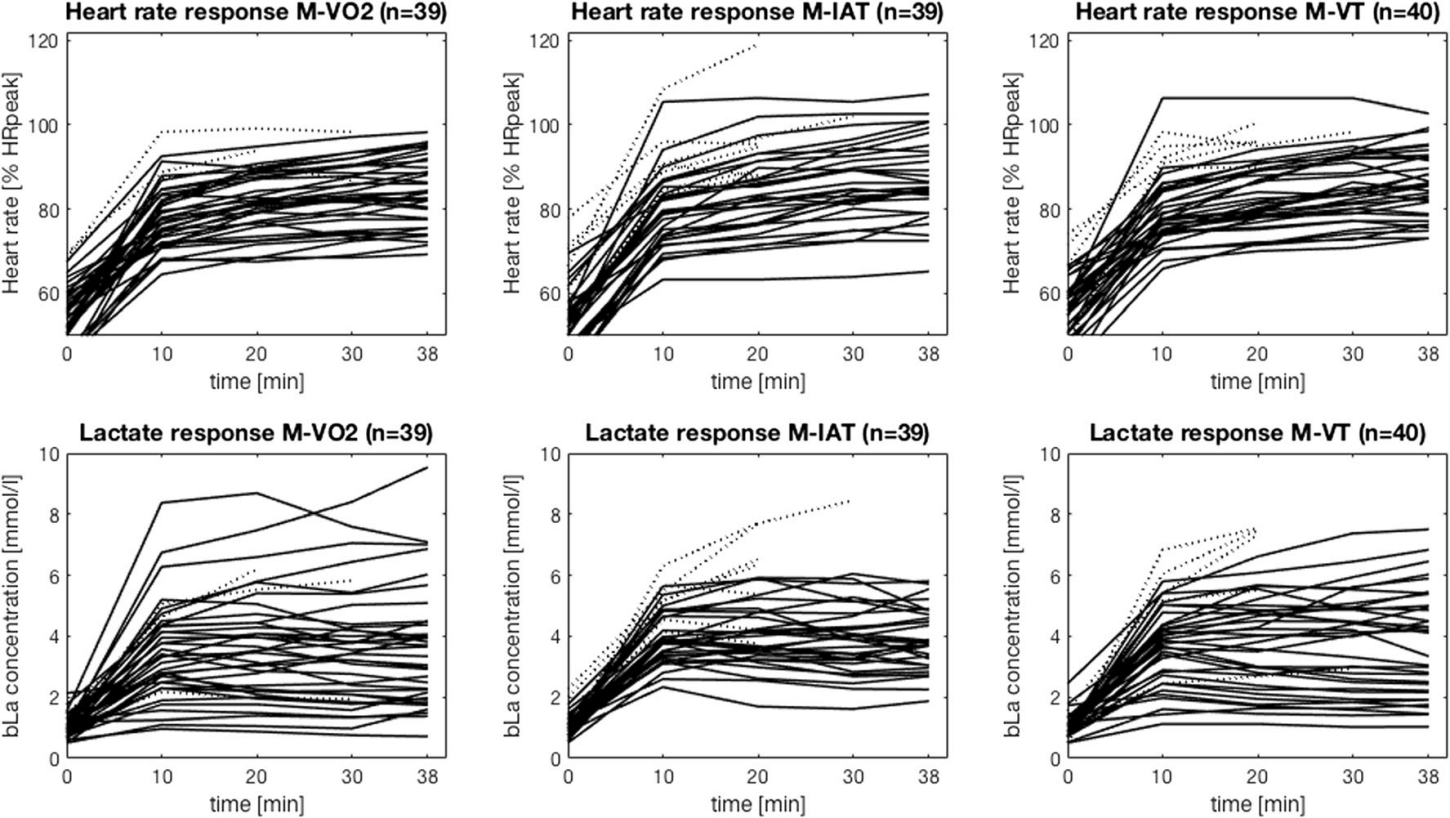
Relative HR (**a**) and bLa (**b**) courses during M-VO_2peak_, M-IAT, and M-VT (from left to right). Intensity during sessions was prescribed as follows: 70% VO_2peak_ (M-VO_2peak_), 97% IAT (M-IAT), and 67% between VT1 and VT2 (M-VT). Dotted lines show courses of participants who terminated the session prematurely. The %HR_peak_ course of one patient is not displayed for M-VO_2peak_ due to a measurement problem

For those who completed all exercise sessions, %HR_peak_, homogeneity of %HR_peak_, mean bLa and the number of participants who reached LASS did not differ between the training sessions (all *p* > .05). However, the variance of bLa during M-IAT was significantly lower compared with M-VO_2peak_ (*p* = .001, *n* = 30) and M-VT (*p* = .022, *n* = 29, Fig. 1). RPE and enjoyment were not different between the training sessions.

## Discussion

In the present study, intensity prescription by means of bLa and ventilatory thresholds was for the first time systematically investigated in breast and prostate cancer survivors after primary therapy and compared with the widely used percentages of VO_2peak_. Our data indicate that within the examined patient population, 70% VO_2peak_, slightly below (97%) IAT, and two-thirds (67%) between VT1 and VT2 were equally suitable to meet the vigorous-intensity zone. As expected, the variance of bLa response was smaller when bLa thresholds were used for intensity prescription, but surprisingly not when ventilatory thresholds were used. In all three training sessions, premature exercise terminations occurred, indicating that intensity was chosen slightly too high.

While prescribed absolute power output was not different between the training sessions, prescribed power output relative to the individual peak power output was higher for M-IAT and M-VT compared with M-VO_2peak_. It must be noted that the used percentage within each intensity prescription method was chosen based on best knowledge and experience to meet the vigorous-intensity zone. This in a sense arbitrary choice naturally affected power output and the resulting physiological and psychological responses. This imbalance should be kept in mind when interpreting the findings. However, they could be leveled out in future by slightly adapting the used percentage within each intensity prescription method: When the early session terminations are additionally considered, 65% VO_2peak_, 90% of IAT, and 60% between VT1 and VT2 (instead of 70%, 97%, and 67%) might be prescriptions for more durable and comparable vigorous-intensity training sessions.

For those participants who completed all training sessions, mean percentage of HR_peak_ corresponded to the vigorous-intensity zone of 77–95% HR_peak_ given by the ACSM for apparently healthy adults [27]. This prescription was shown to be also valid in breast cancer survivors at the end of primary therapy [8]. Mean bLa responses to all three training sessions were nearly 4 mmol L^−1^ which is roughly estimated to correspond to maximal LASS in untrained individuals and thus indicates that the upper limit of the vigorous training zone was met [15, 30]. Altogether, the cardio-metabolic exercise responses indicate that the vigorous-intensity zone was met on average in all three exercise sessions.

Surprisingly, although the cardio-metabolic responses reflected vigorous intensities, RPE reflected moderate intensities according to the ACSM guidelines for apparently healthy adults (RPE 12–13) [27]. Enjoyment was also rated relatively high. However, it has to be considered that all training sessions were supervised in a one-on-one manner and albeit the supervising personal avoided conversations, some participants reported having enjoyed the undivided attention. Furthermore, socially desirable responding could have been an influencing factor. Therefore, subjective exercise responses should be interpreted cautiously.

Considering the homogeneity of physiological strain, the variance of bLa response was lower when bLa thresholds were used for intensity prescription compared with when VO_2peak_ was used. This was in accordance with our hypothesis. Variability in the degree of effort in the CPET might have contributed to the heterogeneity of metabolic strain when intensity was prescribed in percentages of VO_2peak_. However, heterogeneous metabolic responses at given percentages of VO_2peak_ were also found in a previous study with healthy male participants who reached maximal exhaustion during CPET [30]. The authors therefore advised against using %VO_2peak_ for training prescription, although in that study, %VO_2peak_ was not compared with other intensity prescription methods. Surprisingly, variance in bLa response was not as low when ventilatory thresholds were used. This might be attributable to the more challenging determination of ventilatory thresholds as reflected by a lower inter- and intra-evaluator agreement compared with lactate thresholds [31].

In terms of cardiocirculatory strain, there were no statistically significant differences in the variance of heart rate response. However, single participants (outliers) demonstrated heart rates above 100% HR_peak_ only in the sessions prescribed by means of bLa and ventilatory thresholds and not in the session prescribed by means of VO_2peak_ (Fig. 1). Based on this, one might conclude that percentages of VO_2peak_ (or maximal values in general) for intensity prescription are superior to elicit a homogeneous cardiocirculatory strain while bLa thresholds for intensity prescription are superior to elicit a homogeneous metabolic strain. While this appears obvious, confirmatory studies are to the best of our knowledge missing.

Although bLa thresholds have never been used before for intensity prescription in cancer survivors, they represent a general method for individually tailored exercise prescription in high-performance sports [15]. The challenge in the present study was to determine bLa thresholds from a CPET appropriate for cancer survivors. Typically, stepwise incremental exercise protocols with 3-min stages are used and at least five stages are needed for bLa threshold determination [12, 15, 20]. These five stages were easily reached in the present CPET protocol with 1-min stages and the resulting IAT proved useful for intensity prescription—if needed with the above mentioned intensity reduction to 90% IAT. Lactate thresholds constitute the advantage that maximal exhaustion during CPET is not required. This is deemed particularly useful since in the present study 10% and 33% of the conducted CPETs were not considered maximal based on secondary criteria for maximal exhaustion and on the verification test, respectively. Altogether, the IAT derived from a CPET appears useful in cancer survivors which should be further investigated through training intervention studies.

To our knowledge, ventilatory thresholds have not yet been used for prescribing vigorous-intensity exercise in cancer survivors and only three prior studies with cancer survivors did so for prescribing low- to moderate-intensity exercise [17–19]. These studies showed that cardiorespiratory fitness improved after 27 weeks [17], but not after 18 weeks [18, 19] of training at the VT1 performed thrice or twice per week, respectively. Since the intervention groups were compared with non-exercising control groups, no conclusion can be drawn on whether the missing effects were owing to the low exercise stimulus at the VT1 or to the method of intensity prescription itself. Results from studies with healthy participants suggest that moderate to vigorous exercise prescribed by means of ventilatory thresholds elicit superior training adaptations compared with a relative percent concept [14, 32]. More precisely, 100% of the participants who performed 12 weeks of training prescribed by means of ventilatory thresholds demonstrated an improvement in VO_2peak_, whereas only 42% [14] and 60% [32] of those following the same intervention based on percentages of HRR were able to improve their VO_2peak_. Altogether, ventilatory thresholds appear suitable for prescribing also vigorous-intensity exercise in cancer survivors.

### Limitations

The strongest limitation of the present study is the somewhat arbitrary choice of percentages within the prescription methods. This cannot be avoided and although it limits direct comparability of the three training sessions, it does not hamper the conclusions drawn from this study. Furthermore, these findings (including the suggested adaptions of percentages within each intensity prescription method) are prerequisite for implementing the intensity prescription methods into training intervention studies. Another limitation is that threshold concepts might be considered somewhat sophisticated with regard to clinical practice. Yet, as a first approach, we sought to systematically evaluate these methods that are appreciated in elite sports and can be determined without attaining maximal exhaustion in a CPET. In a second approach, it would be interesting to compare these highly objective methods to others that are easier to use, including subjective methods based, e.g., on RPE.

Furthermore, the fact that we did not exclude all patients who had not attained their “true VO_2max_” during CPET might be a limitation, since spending maximal effort during CPET is required for an adequate application of %VO_2peak_ for intensity prescription. However, the validity of secondary criteria for maximal exhaustion has been strongly criticized already for healthy individuals [33, 34] and their applicability has never been assessed in cancer survivors. And, as already mentioned above, we decided to include all patients in the data analyses as this more realistically reflects practice in previous research. Since there was no concordance between the two methods (secondary criteria for maximal exhaustion and verification test) regarding the number of patients who failed to attain maximal exhaustion, it is questionable whether secondary criteria are even useful for cancer survivors. Furthermore, 33% not attaining “real VO_2max_” during CPET raises the question of whether %VO_2peak_ is an appropriate intensity prescription method in cancer survivors, since in the present study, one-third would have exercised at a too low intensity with this method. Even though this assumption is not reflected by the results, it could have more pronounced consequences regarding training responses for interventions with longer durations and/or in larger cohorts.

Finally, it should be noted that sample size was derived from a similar study and not calculated based on expected effects because there were no reference points available. The study has therefore pilot character and further confirmatory research is needed. In addition, the findings are not necessarily transferable to patients with entities other than breast and prostate cancer or to those undergoing anti-cancer treatment.

## Conclusion

In the present study, three training sessions prescribed by means of percentages of VO_2peak_ (reference), blood lactate thresholds, and ventilatory thresholds were compared in terms of durability, physiological, and psychological responses in breast and prostate cancer survivors after primary treatment. There were no significant differences in the number of premature terminations. The vigorous-intensity zone was met on average through all three intensity prescription methods as indicated by cardio-metabolic responses. Blood lactate thresholds appear most suitable for training prescription if a defined metabolic strain is intended because this method elicited the most homogeneous blood lactate response. All three exercise sessions were equally enjoyed and rated as moderate despite their vigorous intensity. Altogether, submaximal thresholds are at least as useful as VO_2peak_ for intensity prescription in breast and prostate cancer survivors after primary treatment. To avoid early session terminations, slightly lower percentages of the reference points might be preferable, e.g., 65% VO_2peak_, 90% IAT, and 60% between VT1 and VT2.
